# MicroRNA‐126 in dogs with immune complex‐mediated glomerulonephritis

**DOI:** 10.1111/jvim.16932

**Published:** 2023-12-20

**Authors:** Ariana D. Cherry, Candice P. Chu, Rachel E. Cianciolo, Jessica A. Hokamp, Sarah A. Jacobson, Mary B. Nabity

**Affiliations:** ^1^ Department of Veterinary Pathobiology, School of Veterinary Medicine & Biomedical Sciences Texas A&M University College Station Texas USA; ^2^ Department of Veterinary Biosciences, College of Veterinary Medicine The Ohio State University Columbus Ohio USA; ^3^ Present address: Niche Diagnostics, LLC Columbus Ohio USA; ^4^ Present address: Zoetis Inc. Columbus Ohio USA

**Keywords:** chronic kidney disease, miR‐126, miR‐182, miR‐21, urine

## Abstract

**Background:**

Most proteinuric dogs with naturally occurring chronic kidney disease have amyloidosis (AMYL), glomerulosclerosis (GS), or immune complex‐mediated glomerulonephritis (ICGN), each with different treatment and prognosis. A noninvasive and disease‐specific biomarker is lacking.

**Hypothesis:**

We hypothesized that the expression pattern of biofluid microRNA (miRNAs and miRs) would correlate with disease progression and categorization.

**Animals:**

Archived serum and urine samples from 18 dogs with glomerular disease and 6 clinically healthy dogs; archived urine samples from 49 dogs with glomerular disease and 13 clinically healthy dogs.

**Methods:**

Retrospective study. Archived biofluid samples from adult dogs with biopsy‐confirmed glomerular disease submitted to the International Veterinary Renal Pathology Service between 2008 and 2016 were selected. Serum and urinary miRNAs were isolated and profiled using RNA sequencing. Urinary miR‐126, miR‐21, miR‐182, and miR‐486 were quantified using quantitative reverse transcription PCR.

**Results:**

When comparing more advanced disease with earlier disease, no serum miRNAs were differentially expressed, but urinary miR‐21 and miR‐182 were 1.63 (95% CI: .86‐3.1) and 1.45 (95% CI: .82‐2.6) times higher in azotemic dogs, respectively (adjusted *P* < .05) and weakly correlated with tubulointerstitial fibrosis (miR‐21: *r* = .32, *P* = .03; miR‐182: *r* = .28, *P* = .05). Expression of urinary miR‐126 was 10.5 (95% CI: 4.1‐26.7), 28.9 (95% CI: 10.5‐79.8), and 126.2 (95% CI: 44.7‐356.3) times higher in dogs with ICGN compared with dogs with GS, AMYL, and healthy controls, respectively (*P* < .001).

**Conclusions and Clinical Importance:**

The miR‐126 could help identify dogs that might benefit from immunosuppressive therapy in the absence of a biopsy. MiR‐21 and miR‐182 are potential markers of disease severity and fibrosis.

**Abbreviations**
95% CI: 95% confidence intervals
AKI: acute kidney injury
AMYL: amyloidosis
AUC: area under curve
CKD: chronic kidney disease
DE: differentially expressed
DN: diabetic nephropathy
GS: glomerulosclerosis
HN: hypertensive nephropathy
ICGN: immune complex‐mediated glomerulonephritis
IgA: IgA nephropathy
MGN: membranous glomerulonephritis
miRNAs: microRNA
miRs: microRNA
MPGN: membranoproliferative glomerulonephritis
qRT‐PCR: quantitative reverse transcription PCR
RNA‐seq: RNA sequencing
ROC: receiver operating characteristic
sCr: serum creatinine
UPC: urine protein : creatinine ratio
USG: urine specific gravity

## 1 INTRODUCTION

Chronic kidney disease (CKD) is an important cause of morbidity and death in all breeds of dogs, commonly caused by underlying glomerular diseases. Over 80% of dogs biopsied because of suspected glomerular disease have amyloidosis (AMYL), glomerulosclerosis (GS), or immune complex‐mediated glomerulonephritis (ICGN).^1^ There is currently no noninvasive or minimally invasive test to reliably diagnose the category of glomerular disease. Renal biopsy and comprehensive pathologic examination are indispensable for diagnosing specific glomerular diagnostic categories and guiding treatment.^2^ Dogs deemed unsuitable for anesthesia or renal biopsy can be empirically managed through immunosuppression; however, immunosuppressive therapy is contraindicated in non‐ICGN glomerular diagnostic categories.^3^

MicroRNAs (miRNAs and miRs) are small, noncoding, highly conserved RNAs that posttranscriptionally regulate gene expression. They play essential roles in governing biological activity in health and disease. To date, 2654 mature miRNA sequences are identified in humans (miRBase 22.1 release). These miRNAs exist in cells and cell‐free biofluids, including serum, plasma, and urine.^4^ Biofluid miRNAs could serve as promising noninvasive biomarkers of CKD onset, diagnosis, progression, therapeutic monitoring, and severity of kidney damage.^5^, ^6^, ^7^

Circulating and urinary miRNAs are identified in humans with glomerular diseases.^6^, ^8^, ^9^, ^10^, ^11^, ^12^, ^13^, ^14^ The most studied diagnostic categories include glomerulonephritis, diabetic nephropathy (DN), IgA nephropathy (IgA), acute kidney injury (AKI), and hypertensive nephropathy (HN), with a focus on miRNAs that affect the pathogenesis of tubulointerstitial fibrosis.^6^, ^8^, ^9^, ^10^, ^11^, ^12^ The most promising and widely studied circulating miRNAs in rodent animal models and humans include miR‐126 (CKD, DN), miR‐21 (AKI, DN), miR‐148 (IgA), and the let‐7b family (IgA).^6^, ^12^ The most promising and widely studied urinary miRNAs include: miR‐21 (AKI, DN), miR‐126 (DN), and miR‐103a (HN).^6^, ^12^ MiR‐21, miR‐29, miR‐103a, and miR‐192 are linked to tubulointerstitial fibrosis and inflammation, indicating their promise as biomarkers of disease progression.^6^, ^12^

Using next‐generation small RNA‐sequencing (RNA‐seq) technology, we aimed to globally characterize miRNAs in urine and serum from clinically healthy dogs and dogs with CKD caused by the 3 most common glomerular disease diagnostic categories (AMYL, GS, and ICGN) at early vs later stages of disease progression (stage A and B). Quantitative reverse transcription PCR (qRT‐PCR) was used to evaluate differentially expressed (DE) urinary miRNAs in a larger cohort of dogs. We hypothesized that unique miRNA signatures would be found in the serum and urine of dogs with each glomerular disease diagnostic category and that these miRNAs might serve as non‐invasive diagnostic markers or therapeutic targets.

## 2 MATERIALS AND METHODS

### 2.1 Animal specimens

In this retrospective study, samples submitted to the International Veterinary Renal Pathology Service at the time of renal biopsy between 2008 and 2016 were used, along with samples from clinically healthy dogs. Archived serum and urine samples were from 18 dogs with glomerular disease (6 from each diagnostic category) and 6 clinically healthy dogs for RNA‐seq (Table S1). For qRT‐PCR, additional archived urine was selected from 49 dogs [AMYL (n = 14), GS (n = 14), and ICGN (n = 21)] and 13 clinically healthy dogs (Table S2). For RNA‐seq, all dogs in the ICGN category had membranous glomerulonephritis (MGN). For qRT‐PCR, the ICGN category consisted of 12 dogs with MGN, 7 dogs with membranoproliferative glomerulonephritis (MPGN), and 2 dogs with mesangioproliferative glomerulonephritis.

For clinically healthy dogs, a physical examination, complete blood count, chemistry panel, urinalysis, and urine protein : creatinine ratio (UPC) were performed to confirm their health status. For dogs with glomerular diseases, the diagnostic category of glomerular disease was confirmed by comprehensive renal biopsy.^15^ Samples were selected based on age (≥1 year), urine sediment within 2 weeks of biopsy without discoloration or cloudiness on gross examination and with <5 WBCs/high‐power field, <100 RBCs/high‐power field, and no bacteriuria, adequate sample volume [serum: ≥1 mL; urine: ≥3 mL (RNA‐seq) or 1 mL (qRT‐PCR)], and nonhemolyzed serum base on a hemolysis score (see Section 2.3).

Within each diagnostic category, CKD dogs were further divided into Stage A (serum creatinine [sCr] <1.4 mg/dL and with biopsy findings limited to minimal to mild tubulointerstitial fibrosis) and Stage B (sCr ≥1.4 mg/dL or sCr <1.4 mg/dL but inappropriately high for the breed and biopsy results demonstrating moderate tubulointerstitial fibrosis). Tubulointerstitial fibrosis was evaluated by a board‐certified anatomic pathologist (REC), scoring 0 to 5, as described.^16^ Each diagnostic category had approximately equal numbers of dogs in each stage.

### 2.2 Sample processing and storage

Urine and serum samples corresponding with the renal biopsy were centrifuged to separate supernatant per standard protocol, shipped overnight on wet ice by the submitting clinician, and stored at −80°C until analysis (1‐6 years). For clinically healthy dogs, uncoagulated blood was allowed to sit at room temperature for 30 to 60 minutes after collection, then centrifuged at 1500*g* for 10 minutes at room temperature to separate serum. Urine was collected via cystocentesis, and urine remaining after the urinalysis was centrifuged at 1000*g* for 10 minutes at 4°C. Serum and urine were aliquoted and stored at −80°C for approximately 3 years until RNA isolation. For clinically healthy dogs, the Texas A&M University Institutional Animal Care and Use Committee (IACUC) approved the protocol (AUP 2012‐021), and client consent was obtained.

### 2.3 RNA isolation

All serum samples were screened for hemolysis by measuring the A385 and A414, using the NanoDrop 2000 (Thermo Fisher Scientific, Wilmington, Delaware) and the following formula: hemolysis score = A414 − A385 + lipemia correlation factor * A385.^17^ Incorporating A385 in the calculation was done to minimize the lipemic interference when measuring A414.^17^ Scores were compared with an in‐house hemolysis score cutoff value generated from a set of 28 grossly nonhemolyzed, leftover clinical samples submitted to the Texas A&M University Veterinary Medical Teaching Hospital (data not shown).

Circulating RNA was isolated from serum by a modified protocol using the Direct‐zol RNA Miniprep Kit (Zymo Research, Irvine, California). For each dog, 1 mL serum was first homogenized with 5 mL QIAzol Lysis Reagent (Qiagen, Germany). The mixture was vortexed, then incubated at room temperature for 5 minutes. Next, 1.2 mL chloroform was added, and lysates were vortexed and incubated at room temperature for 5 minutes, followed by 4°C centrifugation at 13 400*g* for 15 minutes. After centrifugation, the upper aqueous phase was mixed with 4.8 mL 100% ethanol, added to a Zymo‐Spin Column (Zymo Research, Irvine, California), and centrifuged at 12 000*g* for 30 seconds at room temperature. The spin column was then washed twice with Zymo RNA pre‐wash buffer (Zymo Research, Irvine, California), once with Zymo RNA wash buffer (Zymo Research, Irvine, California), and once with 500 μL 80% ethanol, for a total of 4 washes. RNA was eluted with 25 μL 50°C RNase‐free water.

Urinary RNA was isolated from 3 mL (for RNA‐seq) or 1 mL (for qRT‐PCR) urine supernatant from each dog using the Qiagen exoRNeasy Serum/Plasma Maxi Kit (Qiagen, Germany). The manufacturer's protocol was followed up to the point of adding QIAzol Lysis Reagent (Qiagen, Germany). The subsequent steps were identical to the serum isolation protocol, except that the RNeasy MinElute Spin Columns (Qiagen, Germany) were washed 3 times: once with Buffer RWT (Qiagen, Germany) and twice with Buffer RPE (Qiagen, Germany). RNA was eluted with 25 μL 50°C RNase‐free water.

### 2.4 Small RNA sequencing and data analysis

RNA samples were measured using the Fragment Analyzer High Sensitivity RNA Analysis Kit (Advanced Analytical Technologies, Inc., Ankeny, Iowa). The Texas A&M University Genomics Core Lab generated a cDNA library using the NEXTflex Small RNA Library Prep Kit (Bio Scientific Corp, Austin, Texas). Under a 50 base‐pair, single‐end setting, all 48 cDNA libraries were multiplexed and sequenced in parallel on 3 lanes of a flow cell in an Illumina Genome Analyzer (HiSeq 2500v4) to minimize technical variation and ensure sufficient data output.

Preprocessing of raw reads (fastq files) included removal of the 3′ adapter sequence (TGGAATTCTCGGGTGCCAAGG), trimming of the first and last 4 bases from the adapter‐clipped reads (as recommended by the manufacturer),^18^ filtering out reads fewer than 16 base‐pairs to prevent false degraded RNA or adapter dimers,^19^ and removal of low‐quality reads (quality score <30). Untrimmed raw reads were discarded as they were unlikely to be miRNAs based on read lengths. FASTX‐Toolkit (version 0.0.14) was used to transform the fastq format into collapsed fasta files as proper inputs for CPSS 2.0 (https://mcg.ustc.edu.cn/bsc/cpss/).^20^ Default settings along with the canine genome (*Canis familiaris*, CanFam 3.1) and microRNA annotation in miRBase (release 21) were used for analysis. The DESeq2 package in R was used to identify DE miRNAs.^21^ For multiple testing, Wald test *P*‐values were corrected to the false discovery rate (adjusted *P*‐values) by the Benjamini‐Hochberg procedure. An adjusted *P*‐value <.05 was set to select DE miRNAs robustly. NormFinder (updated January 2015) was applied to the read count table to identify candidate miRNAs for internal controls for qRT‐PCR based on the RNA‐seq data,^22^ and we identified urinary miR‐151 and miR‐28 as the most appropriate internal controls (Table S3). These miRNAs were not identified as DE miRNAs in any given pair of comparisons in this study.

### 2.5 qRT‐PCR and data analysis

Based on sequencing data and preliminary qRT‐PCR results (Figures S1 and S3), urinary miR‐126, miR‐21, miR‐182, and miR‐486 were selected as target miRNAs, along with miR‐151 and miR‐28 as reference miRNAs. The miRNA primers were acquired through GeneGlobe (Qiagen, Germany). RNA was reverse transcribed into cDNA using the T100 Thermocycler (Bio‐Rad Laboratories, California) and MiRCURY LNA RT Kit (Qiagen, Germany). The cDNA was diluted using RNAse‐free water at a 1:15 ratio and incorporated in the 10 μL PCR reaction using the miRCURY LNA SYBR Green PCR kit (Qiagen, Germany). The epMotion 6000 (Eppendorf, Massachusetts) was used for plate set‐up to decrease pipetting errors, replicate, and plate differences. The PCR was run on 384‐well plates using the CFX384 Touch Real‐Time PCR Detection System (Bio‐Rad Laboratories, California). Each sample was run in triplicate to account for technical variability and interplate calibrators. A standard curve for each miRNA and controls (NRT, NTC, SYBR neg, and water) were also included. The mean expression among the triplicates was used for analysis. If there were inconsistencies in the amplification curves, primer dimers were ruled out by running the PCR products on 1% agarose gels.

The mean, SD, coefficient of variation, and amplification efficiency for each miRNA were evaluated, and qbase+ was used to normalize the expression of target miRNAs to the reference miRNAs, calibrate expression differences between plates, and remove samples that did not meet QC requirements. Samples with a Cq difference >1.0 between the technical replicates and samples from any plates with miRNA amplification efficiencies <80% were excluded from the analysis. Mann‐Whitney tests and 1‐way ANOVA were used to determine significant differences in log_10_ normalized mean expression between diagnostic categories (miR‐126) and clinical stage (miR‐21, miR‐182, miR‐486). Mann‐Whitney pair‐wise nonparametric tests were used to evaluate miRNAs with non‐normal distributions, while 1‐way ANOVA was used to evaluate miRNAs with normal distribution. The diagnostic performance of miRNAs as biomarkers using qRT‐PCR was evaluated with the Wilson/Brown receiver operating characteristic (ROC) curve analysis. Area under the curve (AUC), 95% confidence intervals (95% CI), and cut points were calculated using non‐parametric ROC methods. AUCs were considered statistically significant when *P* < .05.

The Shapiro‐Wilk goodness of fit test assessed the normality of clinical variables (age, sCr, UPC, urine specific gravity [USG], and fibrosis). Differences in clinical variables among diagnostic categories and stages were performed using Mann‐Whitney pair‐wise nonparametric tests. Correlations between clinical variables and target miRNAs were evaluated using Spearman correlations, with *P* < .05 considered significant. All analyses were performed in GraphPad Prism9 (GraphPad Software, San Diego, California) except for the AUC analysis, which used STATA17 (StataCorp LLC, College Station, Texas).

## 3 RESULTS

### 3.1 Clinical variables for each diagnostic category and clinical stage

**FIGURE 1:**
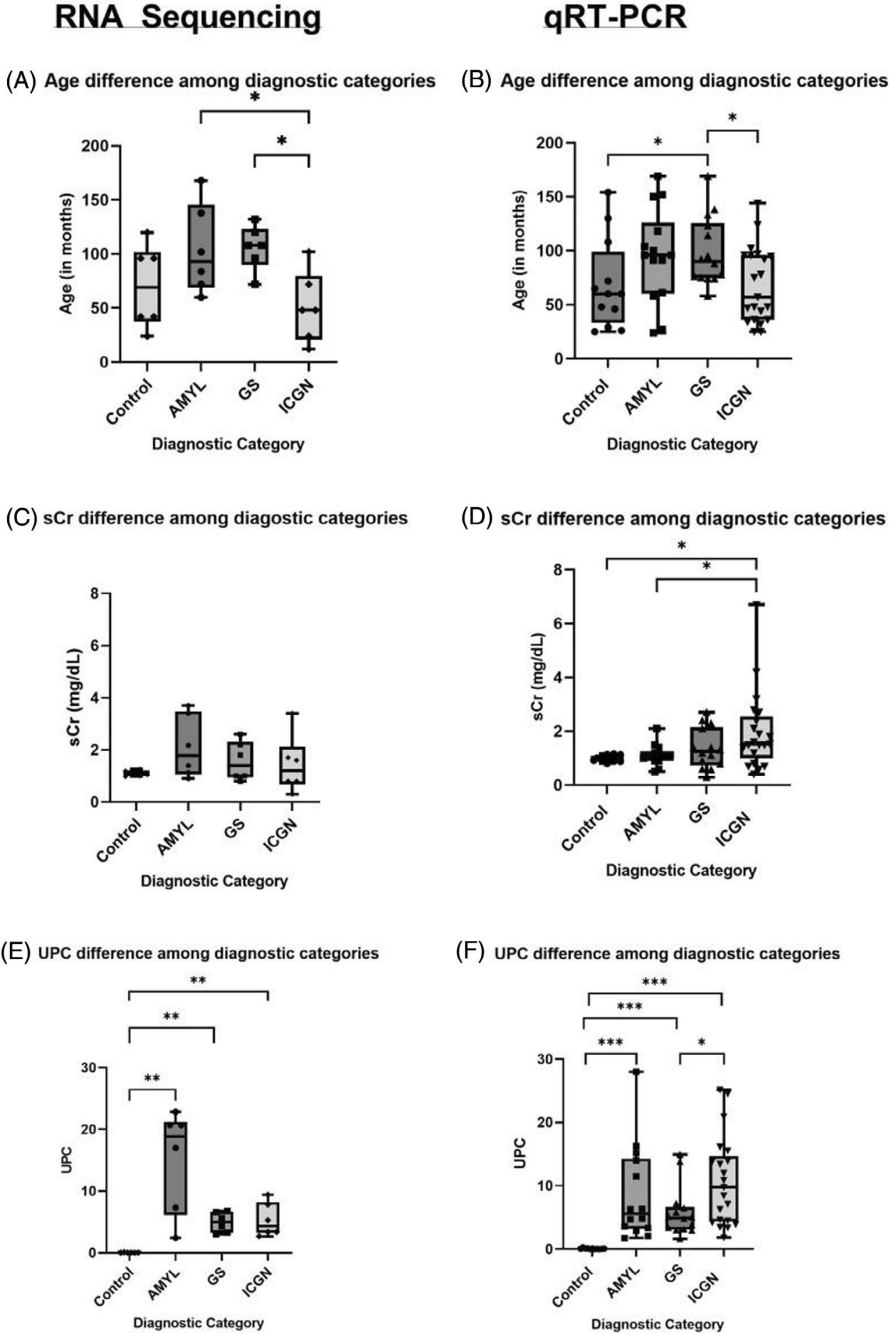
Clinical variables across diagnostic categories from dogs used in RNAseq and qRT‐PCR. Boxplots of age, serum creatinine (sCr), and urine protein : creatinine ratio (UPC) distribution for dogs whose samples were used for the small RNA‐seq analysis (A,C,E) vs the qRT‐PCR dataset (B,D,F) among different diagnostic categories. The median expression and individual values are displayed on the boxplot; bars represent the upper, middle, and lower quartiles. The Mann‐Whitney *U* test was used in a pair‐wise manner to compare the continuous variables of each category. *P < .05; **P < .01; ***P < .001. AMYL, amyloidosis; GS, glomerulosclerosis; ICGN, immune complex‐mediated glomerulonephritis.

The median age was significantly lower for the ICGN category compared with AMYL (*P* < .05) and GS (*P* < .05) for dogs in the RNA‐seq cohort and significantly lower compared with GS (*P* < .05) in the qRT‐PCR cohorts. The median age of dogs in the GS category was significantly higher than controls in the qRT‐PCR cohort (Figure 1A,B, *P* < .05). No significant difference in median sCr was observed among diagnostic categories for the RNA‐seq cohort; however, in the qRT‐PCR cohort, sCr was significantly higher in dogs with ICGN compared with dogs with AMYL and controls (Figure 1C,D, *P* < .05). UPC was significantly higher in dogs with ICGN compared with dogs with GS, and UPC was significantly higher in all diagnostic categories compared with controls (Figure 1E,F; *P* < .05). As expected, based on our study design, sCr was significantly higher in stage B dogs than in stage A dogs and controls (Figure S2; *P* < .05). UPC was also significantly higher in stage A and B dogs compared with controls (Figure S2; *P* < .05). There was no difference in age between stage A and B dogs (Figure S2). For complete clinical information, refer to Tables S1 and S2.

### 3.2 Small RNA‐seq: Differentially expressed (DE) serum and urinary miRNAs

Forty‐eight RNA samples were used for RNA‐seq, 24 each from serum and urine. One serum sample representing stage B ICGN was excluded for low reads (<5 million; Table S3). Sequenced serum samples averaged 6.5 million reads and a 97.6% genome mapping rate, with 1.6 million reads mapped to miRNAs. Urine samples had an average of 6.9 million reads and a 90.1% genome mapping rate, with 79 019 reads mapped to miRNAs. On average, 167 and 88 miRNAs with at least 10 mapped reads were detected in serum and urine samples, respectively.

**FIGURE 2:**
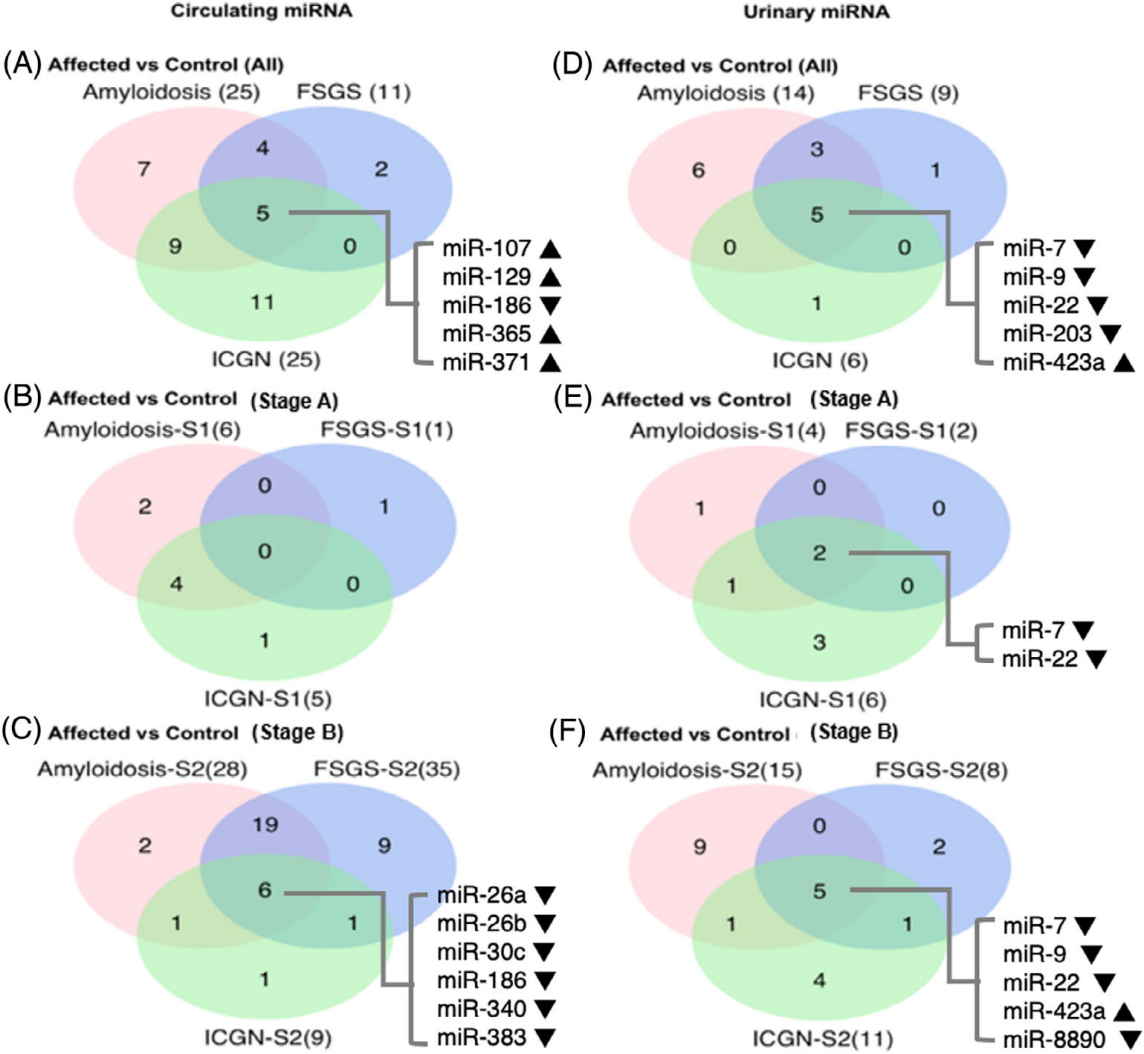
Differentially expressed (DE) serum and urinary miRNAs identified in CKD dogs (based on glomerular disease category) vs controls. Dogs with CKD were compared with controls regardless of stage (A,D), in Stage A (B,E), and Stage B (C,F). The total number of DE miRNAs are shown in parenthesis following each disease category. Only those DE miRNAs that were also differentially expressed when comparing all dogs with CKD with controls in the respective group are included. Differential expression was based on an absolute fold change >2 and an adjusted *P*‐value <.05 (S1: Stage A CKD; S2: Stage B CKD; AMYL [amyloidosis], GS [glomerulosclerosis] and ICGN [immune complex‐mediated glomerulonephritis]; ▲: upregulation in affected dogs, ▼: downregulation in affected dogs).

Overall, 38 serum and 16 urinary miRNAs were DE in CKD dogs vs controls (Figure 2). In each diagnostic category, CKD dogs were compared with controls regardless of disease stage (Figure 2A,D). CKD dogs were further divided into stages A (Figure 2B,E) and B (Figure 2C,F) and compared with controls. Comparing stage A dogs with controls, 8 serum and 7 urinary DE miRNAs were detected, while 39 serum and 22 urinary DE miRNAs were discovered comparing stage B dogs with controls. Regardless of diagnostic category or stage, 5 serum miRNAs (miR‐107, miR‐129, miR‐186, miR‐365, and miR‐371) and 5 urinary miRNAs (miR‐7, miR‐9, miR‐22, miR‐203, and miR‐423a) were DE in dogs with glomerular disease when compared with controls (Figure 2A,D). Downregulated serum miR‐186 was also among the 6 common DE miRNAs among all 3 diagnostic categories when comparing stage B CKD dogs to controls (Figure 2C). Downregulated urinary miR‐7 and miR‐22 were the only 2 common DE miRNAs among all 3 diagnostic categories identified in stage A dogs compared with controls. They also comprised 2 of the 5 DE urinary miRNAs in stage B dogs compared with controls (Figure 2E,F). Lists of all serum and urinary DE miRNAs are provided in Tables S5 and S6.

### 3.3 Small RNA‐seq: DE miRNAs associated with disease progression

When all CKD dogs were combined, regardless of the glomerular diagnostic category, no serum miRNAs were DE between stages A and B. However, 3 urinary DE miRNAs were identified between stage A and B dogs, including upregulated miR‐182 and miR‐21 and downregulated miR‐486 (Figure S3). The expression of urinary miR‐486 was significantly decreased in stage B dogs compared with both stage A dogs and controls (Figure S3).

### 3.4 Small RNA‐seq: Glomerular disease‐specific urinary miRNAs

We aimed to identify biofluid‐derived miRNAs exclusively differentially expressed in specific diagnostic categories of glomerular diseases in dogs. No serum miRNAs were differentially expressed among the different diagnostic categories when including all dogs in each category, regardless of disease stage.

**FIGURE 3:**
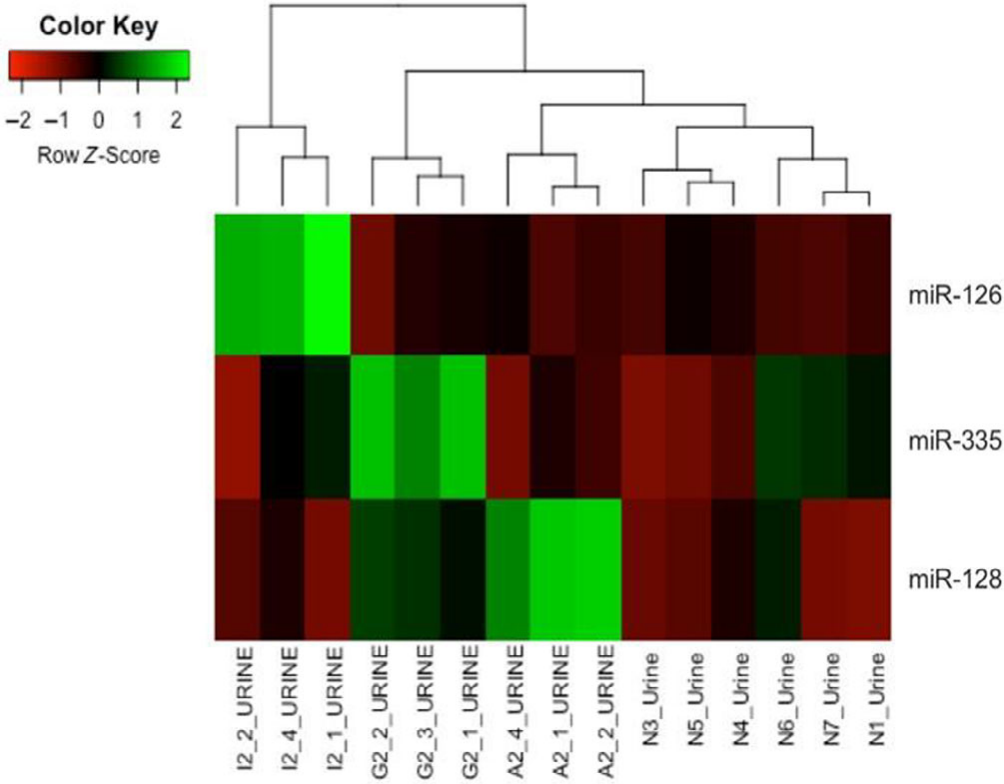
Heatmap and clustering analysis of urinary miR‐126, miR‐335, and miR‐128 in dogs with glomerular diseases and controls. Stage B dogs were included in this analysis. Each column of the heatmap represents a dog, and each row represents a miRNA. Color intensity increases proportionally to the magnitude of the change. As denoted by the green hue (high row *Z*‐score), the expression levels of miR‐126, miR‐335, and miR‐128 are significantly higher in azotemic dogs diagnosed with I2 (ICGN), G2 (GS), and A2 (AMYL), respectively. AMYL, amyloidosis; GS, glomerulosclerosis; ICGN, immune complex‐mediated glomerulonephritis.

When comparing disease stages within each diagnostic category, there was upregulation of serum miR‐1836 for AMYL in several comparisons (Table 1). The annotation of cfa‐miR‐1836 overlaps with snoRNA 20 (SNORA20)^23^; therefore, miR‐1836 was excluded from further analysis. For stage A, 3 serum miRNAs (miR‐335, miR‐101, and miR‐32) and 5 serum miRNAs (miR‐320, miR‐99b, miR‐218, miR‐335, and miR‐485) were differentially expressed in ICGN compared with AMYL and GS, respectively. No urinary DE miRNAs were identified among stage A samples. In stage B dogs, serum miR‐350 and miR‐374a were differentially expressed in GS compared with AMYL and ICGN, respectively, while 5 urinary DE miRNAs were detected in at least 1 pair of comparisons among the 3 glomerular diseases. Notably, the distinctive expression of urinary miR‐126, miR‐335, and miR‐128 could correctly group stage B dogs into ICGN, GS, or AMYL (Figure 3). Based on preliminary qRT‐PCR results, miR‐126 appeared most promising and was therefore selected for further evaluation as a marker of ICGN.

**TABLE 1:** Differentially expressed miRNAs identified in each glomerular disease category in Stage A and B dogs.

Serum					
Stage A			Stage B		
miRNA	Fold change	Adjusted *P*‐value	miRNA	Fold change	Adjusted *P*‐value
*AMYL* vs *GS*					
N/A			miR‐1836^a^	−3 726 076	.00002
			miR‐350	91.6	.03
*AMYL* vs *ICGN*					
miR‐335	86.7	.01	miR‐1836^a^	−5 769 001	.0002
miR‐101	3.67	.05			
miR‐1836^a^	23 852	.05			
miR‐32	14.9	.05			
*GS* vs *ICGN*					
miR‐320	−4.30	.003	miR‐374a	−5.86	.02
miR‐99b	−3.18	.04			
miR‐218	6.23	.05			
miR‐335	42.0	.05			
miR‐485	−19.0	.05			

Urine					
Stage A			Stage B		
miRNA	Fold change	Adjusted *P*‐value	miRNA	Fold change	Adjusted *P*‐value
*AMYL* vs *GS*					
N/A			miR‐335	−9.61	.013
			miR‐8904b	20.8	.013
*AMYL* vs *ICGN*					
N/A			miR‐126	−10.9	.013
			miR‐128	4.15	.013
			miR‐143	−7.02	.045
*GS* vs *ICGN*					
N/A			miR‐8904b	−24.3	.007
			miR‐126	−26.3	.047

*Note*: Each section denotes a disease sample type (Serum or Urine), disease stage (Stage A or Stage B) and the pair of comparisons (AMYL vs GS, AMYL vs ICGN, and GS vs ICGN).

Abbreviations: AMYL: amyloidosis; GS, glomerulosclerosis; ICGN, immune complex‐mediated glomerulonephritis; N/A, no differentially expressed miRNA detected. For example, no differentially expressed miRNAs were detected when comparing serum samples from stage A AMYL dogs to stage A GS dogs.

^a^ miR‐1836: See main text for the description of cfa‐miR‐1836.

### 3.5 qRT‐PCR of urinary miR‐126, miR‐21, miR‐182, and miR‐486

**FIGURE 4:**
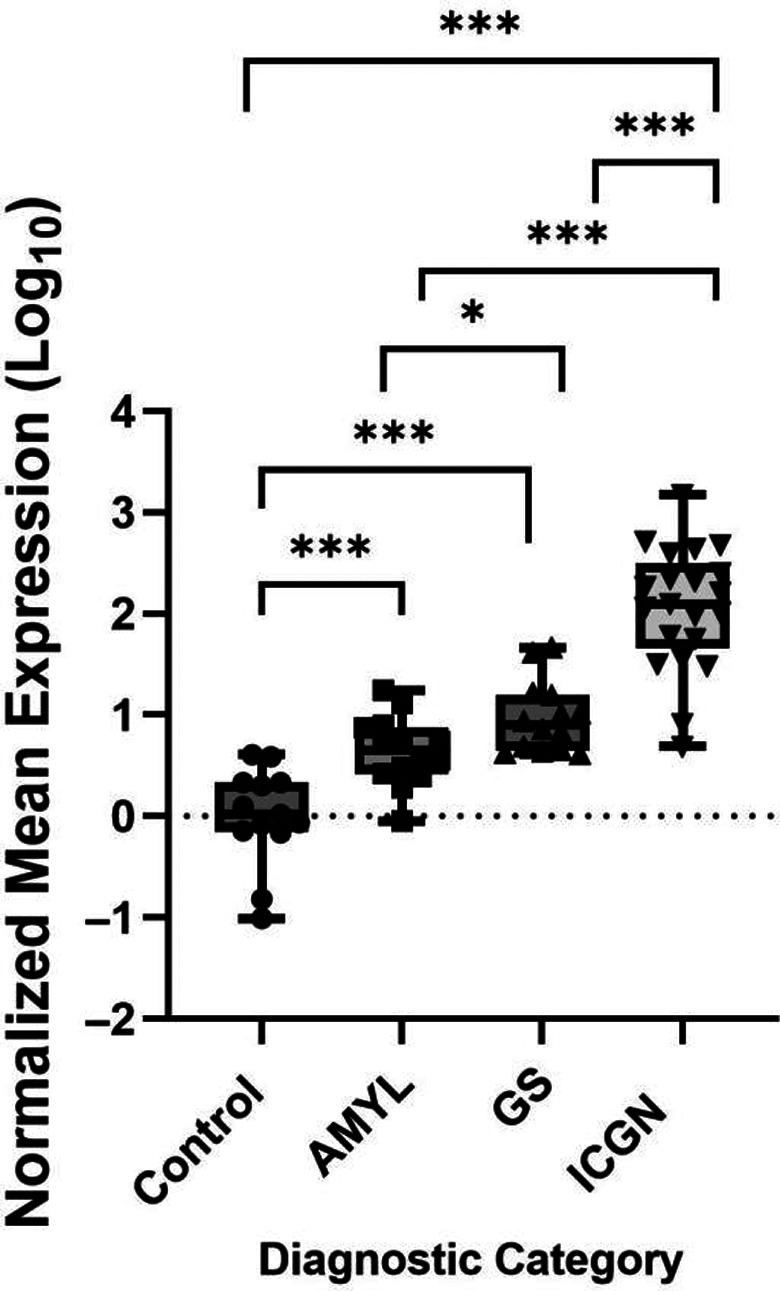
Normalized mean expression of urinary miR‐126 based on qRT‐PCR in clinically healthy dogs (Control n = 14), dogs with amyloidosis (AMYL, n = 14), glomerulosclerosis (GS n = 19), and immune complex‐mediated glomerulonephritis (ICGN n = 19). The normalized mean expression of miR‐126 was transformed to Log10 and plotted by diagnostic category. The median expression and individual values are displayed on the boxplot. Bars represent the upper, middle, and lower quartiles. Tukey for multiple testing was used to test for differential expression. *P < .05; **P < .01; ***P < .001.

The qRT‐PCR confirmed that the normalized mean expression of urinary miR‐126 was significantly higher in dogs with ICGN compared with AMYL, GS, and controls (Figure 4), being 10.5 times higher than in dogs with GS (95% CI: 4.1‐26.7; *P* < .001) and 28.9 times higher compared with dogs with AMYL (95% CI: 10.5‐79.8; *P* < .001). Urinary miR‐126 expression was also 2.8 times higher in dogs with GS compared with dogs with AMYL (95% CI: 1.0‐7.6; *P* < .05). No significant difference was observed within ICGN dogs between stage A and B or the ICGN subcategories (not shown). Urinary miR‐126 expression was weakly to moderately correlated with sCr (*r* = .330, *P* = .008), UPC (*r* = .493, *P* < .001), and fibrosis (*r* = .39, *P* = .006; Table S7).

**FIGURE 5:**
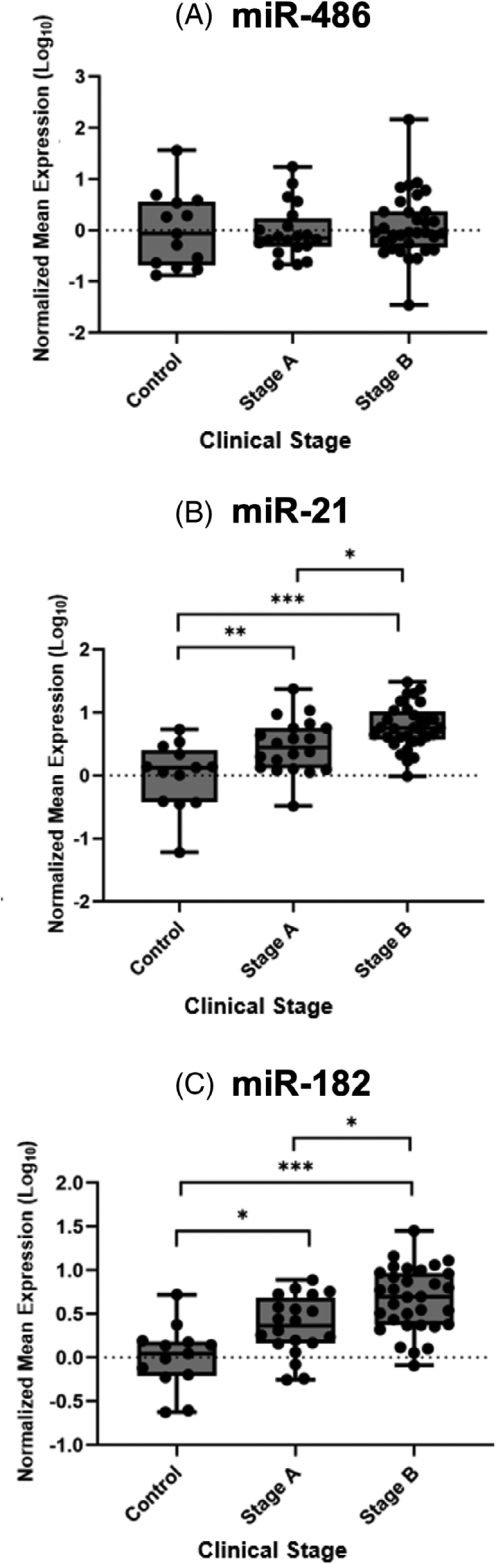
Normalized mean expression of urinary miR‐486 (A) miR‐21 (B), and miR‐182 (C) in clinically healthy (Control n = 13), Stage A (n = 21), and Stage B (n = 32) dogs. The median expression and individual values are displayed on the boxplot. Bars represent the upper, middle, and lower quartiles. The Mann‐Whitney *U* test was used in a pair‐wise manner to compare the continuous variables of each category. *P < .05; **P < .01; ***P < .001.

The normalized mean expression of urinary miR‐21 and miR‐182 differed between stages and were 1.63 (95% CI: .86‐3.1) and 1.45 (95% CI: .82‐2.6) times higher, respectively, in stage B vs stage A dogs (Figure 5). Despite being differentially expressed based on RNA‐seq, there was no difference in miR‐486 between stage A and B dogs based on qRT‐PCR (Figure 5). MiR‐21 was moderately correlated with UPC (*r* = .42, *P* < .001) and weakly correlated with fibrosis (*r* = .32, *P* = .03; Table S7). Likewise, miR‐182 was weakly correlated with UPC (*r* = −.25, *P* = .05) and fibrosis (*r* = .28, *P* = .05; Table S7). No significant correlations with clinical variables were observed for miR‐486. Fibrosis was weakly correlated with clinical stage (*r* = .39, *P* = .006; Table S7), but no significant difference in fibrosis score between Stage A and B dogs was seen (Figure S4). There was also no difference in the normalized mean expression of miR‐21 or miR‐182 between dogs with minimal, mild, and moderate fibrosis (Figure S4).

### 3.6 Diagnostic evaluation of urinary miR‐126

**FIGURE 6:**
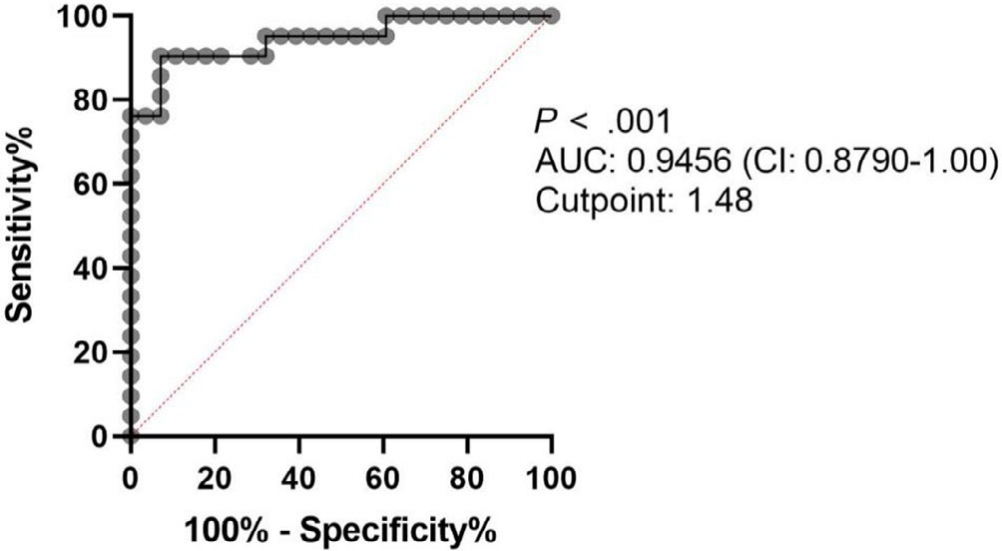
Receiver operating characteristic (ROC) curve for miR‐126 comparing dogs with immune complex‐mediated glomerulonephritis (ICGN) vs dogs with amyloidosis (AMYL) and glomerulosclerosis (GS). The area under the curve (AUC) and its confidence interval are shown, using a cut‐point of 1.48 Log_10_ miRNA expression, sensitivity is 90% (95% CI: 71‐98%), and specificity is 92% (95% CI: 77‐99%) for ICGN (*P* < .001).

ROC curve analysis was performed to assess the ability of urinary miR‐126 to distinguish between ICGN and non‐ICGN (AMYL and GS; Figure 6). With a normalized mean expression and Log_10_ cut‐point of 1.48, the AUC for miR‐126 to differentiate dogs with ICGN vs non‐ICGN was 0.95 (95% CI: 0.88‐1.0, *P* < .001), with a sensitivity and specificity of 90% (95% CI: 71%‐98%) and 92% (95% CI: 77%‐99%), respectively.

## 4 DISCUSSION

In our study, we identified miR‐126 as a promising biomarker for the identification of ICGN. In our RNA‐seq data, the upregulation of urinary miR‐126, miR‐355, and miR‐128 was exclusively seen in Stage B dogs diagnosed with ICGN, GS, and AMYL, respectively (Figure 3). In particular, we demonstrated a significant increase in urinary miR‐126 in Stage B dogs with ICGN compared with GS and AMYL (Figure 4). This was further verified by qRT‐PCR, where a significant increase was found in dogs with both MGN and MPGN, regardless of disease stage. MiR‐126 had high sensitivity (90%) and specificity (92%) for ICGN compared with other glomerular diagnostic categories, correctly classifying 92% of cases. These findings support that urinary miR‐126 could be used clinically as a non‐invasive test to identify ICGN in dogs with suspected glomerular disease, using a normalized Log_10_ mean expression cut‐point value ≥1.48. As ICGN is the most common category of glomerular disease in dogs biopsied for suspicion of glomerular disease^1^ and immunosuppressive therapy is typically recommended, a non‐invasive test for ICGN would be particularly helpful.

In human CKD patients, early studies on the expression of biofluid‐derived miRNAs provided limited information due to the low numbers of miRNAs screened^13^, ^14^ or identification of miRNAs with low fold changes (<2‐fold).^24^ With the development of next‐generation sequencing, recent studies have provided more extensive and robust data.^25^ Small RNA‐seq studies are limited in dogs with CKD,^26^ and in both human and veterinary medicine, studies investigating biofluid‐derived miRNAs to distinguish between different categories of renal disease are lacking.

MiR‐126 is an endothelial‐specific miRNA important to vascular integrity. While no published literature currently exists regarding the expression of miR‐126 in ICGN, there is increased expression of urinary miR‐126 in patients with type 2 diabetes mellitus and DN compared with patients without DN.^27^ MiR‐126 is increased in urine‐derived extracellular vesicles released from renal epithelial cells undergoing mesenchymal transition and is associated with increased albuminuria, fibrosis, and progression in patients with DN.^28^ In contrast, our data and studies on humans with DN documented a significant decrease in circulating miR‐126 expression in dogs and patients with CKD when compared with controls (Table S5).^29^, ^30^ MiR‐126 was enriched in endothelial progenitor cell‐derived extracellular vesicles and is thought to be responsible, in part, for their protective effect against ischemic AKI.^31^ In humans, circulating miR‐126 is upregulated in autoimmune diseases such as multiple sclerosis and rheumatoid arthritis.^32^, ^33^, ^34^ Kidney‐derived miR‐126 is downregulated in an ischemic AKI model using human kidneys donated after cardiovascular death and is associated with reduced renal function and increased interleukin 6 expression, promoting neutrophil recruitment and fibrosis.^35^ Upregulation of miR‐126 is part of the pathway that leads to increased production of interferons and proinflammatory cytokines in innate immune responses in the lungs of mice and in circulating miR‐126 in humans with asthma.^36^, ^37^, ^38^ The miR‐126 upregulation in these models leads to an inflammatory response and dysregulation of immune cells, like neutrophils and plasmacytoid dendritic cells. Our findings could indicate that miR‐126 plays a role in the immune and inflammatory responses in dogs with ICGN.

In our study, RNA‐seq also identified miR‐335 and miR‐128 as promising urinary biomarkers to categorize GS and AMYL, respectively. In knockout and over‐expression studies, miR‐335 contributes to the accumulation of reactive oxygen species and consequent renal aging.^39^ While the pathophysiological mechanisms of GS are varied and often not entirely understood, pathways involving reactive oxygen species have been postulated.^40^ Several studies found agents that lower reactive oxygen species levels^41^, ^42^ or stimulate superoxide dismutase‐SOD activity^43^ could attenuate GS in murine models. Therefore, miR‐335 might be involved in the pathogenesis of GS in dogs; however, preliminary qRT‐PCR results were discouraging in their ability to distinguish GS from AMYL and ICGN (Figure S1). Overexpression of miR‐128 induces apoptosis in human embryonic kidney cells (HEK293T).^44^ In normal rat kidney cells, in which miR‐128 was overexpressed, the expression of pro‐inflammatory genes was also upregulated, and the MAPK pathway was the top enriched pathway of miR‐128 target genes.^45^

In addition to identifying DE miRNAs in dogs with CKD, we explored biofluid‐derived miRNAs that might indicate disease progression.^46^ Urinary miR‐21 was differentially expressed in Stage B dogs compared with Stage A and control dogs despite the overlaps in the expression. This finding is similar to other studies in dogs with azotemic kidney disease.^26^, ^47^ In our study, miR‐21 expression was positively correlated with UPC and fibrosis (Table S7). Similarly, in dogs with X‐linked hereditary nephropathy, kidney miR‐21 increased with the development of azotemia and is significantly correlated with sCr, UPC, GFR, and histologic lesions (fibrosis, inflammation, and tubular damage) based upon qRT‐PCR and normalization with miR‐16.^47^ More reads were mapped to miR‐21 in dogs with azotemic kidney disease compared with controls in another RNA‐seq study,^26^ and miR‐21 is upregulated in humans with AKI, CKD, and DN.^48^, ^49^, ^50^ MiR‐21 targets and represses SMAD7 and PTEN expression, leading to increased TGF‐B1 activity and fibrosis.^51^ In humans with IgA nephropathy, miR‐21 and fibrosis are highly correlated.^52^ While miR‐21 was weakly correlated with fibrosis in our study, fibrosis scores were not significantly different in the qRT‐PCR cohort between Stage A and B dogs, and miR‐21 was not differentially expressed in dogs with moderate to severe fibrosis when compared with dogs with minimal to mild fibrosis (Figure S4).

Urinary miR‐182 expression was increased in Stage B dogs compared with Stage A and control dogs and was significantly correlated with UPC and fibrosis (Table S7). In a rat renal proximal tubular cell line and an ischemia/reperfusion‐induced rat model, miR‐182 inhibits the TCF7L2/Wnt/B‐catenin pathway, increasing apoptosis which is exacerbated in AKI, supporting its potential involvement in promoting TI damage.^53^ Further, the silencing of miR‐182 alleviates renal injury in rats.^54^ MiR‐182 is upregulated, along with TGF‐B1 and other fibrotic factors in mice with polycystic disease, and silencing of miR‐182 leads to decreases in concentration of downstream fibrotic factors, such as collagen I and IV, while TGF‐B remained high.^55^ These findings suggest that miR‐182 is upregulated in the fibrotic pathway leading to apoptosis and cell death in renal cells. Like miR‐21, urinary miR‐182 was not different in dogs with moderate fibrosis compared with dogs with minimal and mild fibrosis (Figure S4). For both urinary miR‐21 and miR‐182, there was no correlation with sCr, supporting that they are not markers of glomerular filtration rate.

Our RNA‐seq data indicated downregulation of urinary miR‐486 in Stage B dogs compared with Stage A dogs. In humans with DN, serum miR‐486 is downregulated compared with healthy controls and is negatively associated with albuminuria and estimated glomerular filtration rate.^46^ MiR‐486 downregulation is associated with signaling pathways consisting of genes related to CKD pathogenesis, including oxidative stress, inflammation, and apoptosis.^46^ Downregulation of miR‐486 in CKD dogs might play a role in the progression of CKD. The RNA‐seq data were inconsistent with the qRT‐PCR data, which showed no differential expression between Stage A and B dogs. Our data was also inconsistent with a sequencing study in which a pooled urine sample from dogs with kidney disease had more reads mapped to miR‐486 than a pooled control sample.^26^

There were several limitations to our study. While we included samples from dogs with the most common categories of glomerular disease, other glomerular diagnostic categories were not included. Therefore, whether our findings will apply to these other groups, particularly for miR‐126, is unknown. Dogs included in the qRT‐PCR cohort did not have an extensive range of fibrosis observed on biopsy, limiting correlations with fibrosis, although significant correlations were observed with 3 of the 4 miRNAs evaluated.

The retrospective study design resulted in differing pre‐analysis sample storage times. Also, despite providing a standard protocol for sample processing to submitting veterinarians, identical sample processing could not be guaranteed. The stability of the miRNAs is unknown and might be affected by processing, storage time and conditions, and freeze‐thaw cycles.

## 5 CONCLUSIONS

Our study identified serum and urinary miRNAs that differ between clinical stage and diagnostic categories of glomerular disease. In particular, urinary miR‐126 was upregulated in dogs with ICGN compared with dogs with AMYL and GS. Therefore, it has the potential to be a non‐invasive biomarker to identify dogs that would benefit from immunosuppressive therapy in the absence of a renal biopsy. Furthermore, urinary miR‐21 and miR‐182 could be a marker of disease severity and fibrosis.

## CONFLICT OF INTEREST DECLARATION

Authors declare no conflict of interest.

## OFF‐LABEL ANTIMICROBIAL DECLARATION

Authors declare no off‐label use of antimicrobials.

## INSTITUTIONAL ANIMAL CARE AND USE COMMITTEE (IACUC) OR OTHER APPROVAL DECLARATION

Protocol approved by the IACUC of the Texas A&M University (AUP 2012‐021).

## HUMAN ETHICS APPROVAL DECLARATION

Authors declare human ethics approval was not needed for this study.

## Supporting information


**Data S1.** Supporting Information.

**Table S1.** Global urinary microRNA profiling of canine glomerular diseases revealed elevation of miRNA 126 in dogs with immune complex‐mediated glomerulonephritis—individual dog data, descriptive stats.

**Table S2.** Global urinary microRNA profiling of canine glomerular diseases revealed elevation of miRNA 126 in dogs with immune complex‐mediated glomerulonephritis—inclusion criteria, qRT‐PCR clinical parameters, descriptive stats.

**Table S3.** Global urinary microRNA profiling of canine glomerular diseases revealed elevation of miRNA 126 in dogs with immune complex‐mediated glomerulonephritis—internal controls.

**Table S4.** Global urinary microRNA profiling of canine glomerular diseases revealed elevation of miRNA 126 in dogs with immune complex‐mediated glomerulonephritis—fibrosis score.

**Table S5.** Global urinary microRNA profiling of canine glomerular diseases revealed elevation of miRNA 126 in dogs with immune complex‐mediated glomerulonephritis—circulating miRNAs.

**Table S6.** Global urinary microRNA profiling of canine glomerular diseases revealed elevation of miRNA 126 in dogs with immune complex‐mediated glomerulonephritis—urinary miRNAs.

**Table S7.** Global urinary microRNA profiling of canine glomerular diseases revealed elevation of miRNA 126 in dogs with immune complex‐mediated glomerulonephritis—Spearman correlations.
