# Supplementary material for: MicroRNA‐126 in dogs with immune complex‐mediated glomerulonephritis
Source: J Vet Intern Med. 2023 Dec 20;38(1):216–27. doi: 10.1111/jvim.16932 (PMC10800198; doi:10.1111/jvim.16932)
Supplement: Supplementary file 1 — Data S1. Supporting Information. [file JVIM-38-216-s002.docx]

**MicroRNA‐126 in dogs with immune complex‐mediated glomerulonephritis**
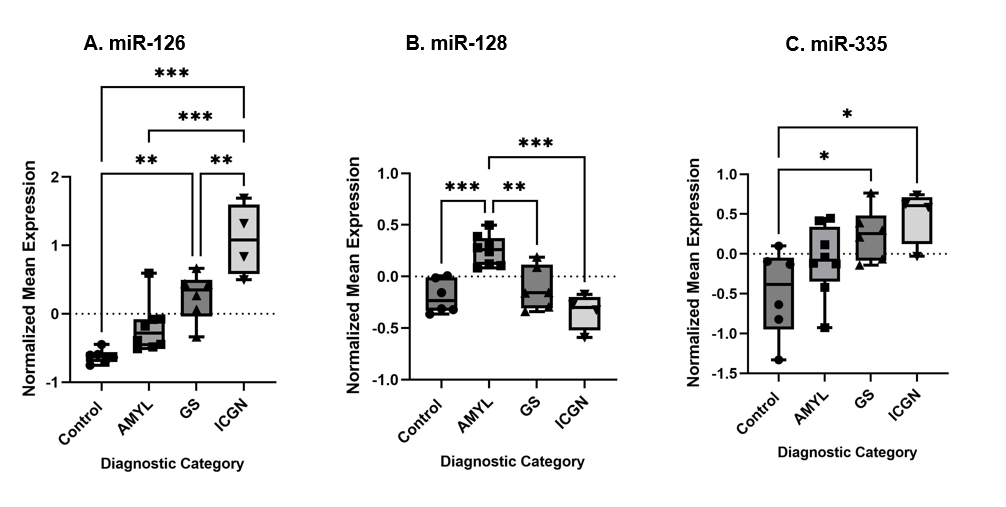


Supplementary Figure S1: Normalized mean expression of miR-126, miR-128 and miR-335 based on preliminary qRT-PCR analysis in clinically healthy dogs (Control, n=6), dogs with amyloidosis (AMYL, n=8), glomerulosclerosis (GS, n=6), and immune complex-mediated glomerulonephritis (ICGN, n=4). The median expression and individual values are displayed on the boxplot. Bars represent the upper, middle, and lower quartiles. Normalized mean expression of each miR was transformed to Log10 and plotted by diagnostic category. Tukey for multiple testing was used to test for differential expression. **P* < .05; ***P* < .01; ****P* < .001.


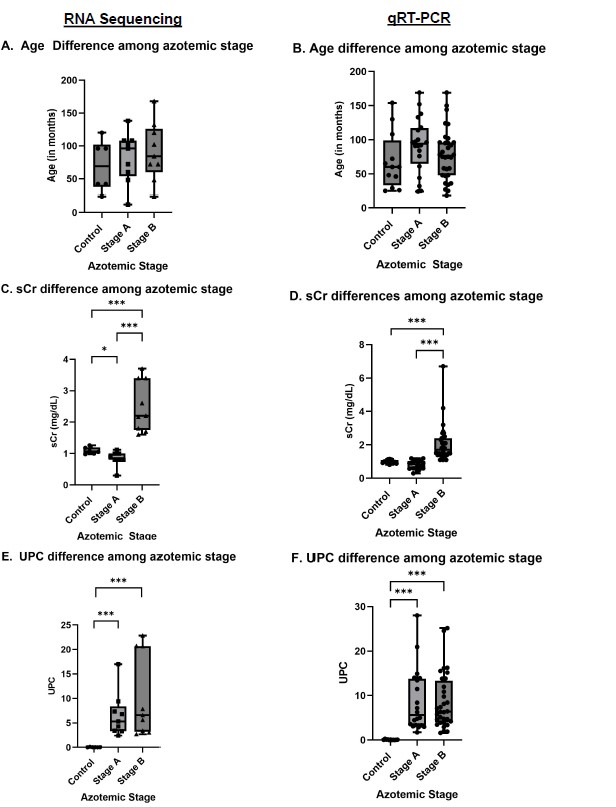


Supplementary Figure S2. Clinical parameters across in Stage A and Stage B dogsused in RNAseq and qRT-PCR analysis. The median expression and individual values are displayed on the boxplot. Bars represent the upper, middle, and lower quartiles. Boxplots of age, serum creatinine (sCr), and urine protein: creatinine ration (UPC) distribution for dogs whose samples were used for the small RNA-seq analysis (A, C, E) versus the qRT-PCR dataset (B , D, F) among different azotemic statuses. Stage A: sCr < 1.4 mg/dl or appropriately low for the breed and with biopsy findings limited to minimal to mild tubulointerstitial (TI) fibrosis. Stage B: 1.4 ≤ sCr ≤ 5 mg/dl) or sCr < 1.4 mg/dl but inappropriately high for the breed and biopsy results demonstrating significant TI fibrosis (moderate to severe). Mann Whitney U test was used in a pair-wise manner to compare the median of each category. *P < .05; **P < .01; ***P < .001.


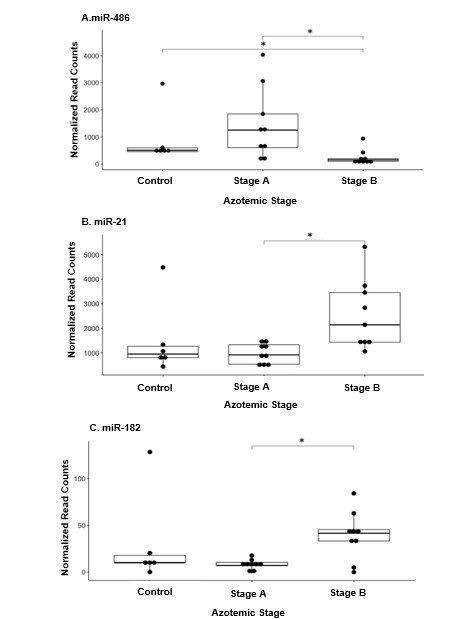


Supplementary Figure S3: Normalized read counts for urinary DE miRs identified between control, Stage A and Stage B CKD dogs using RNA-seq. Individual data points are plotted for each stage, and box plots show the median and upper and lower quartiles. Tukey for multiple testing was used (**P* < .05). Read counts were significantly lower for miR-486 (A) and higher for miR-21 (B), and miR-182 (C) in Stage B dogs compared with Stage A dogs and, for miR-486, controls.


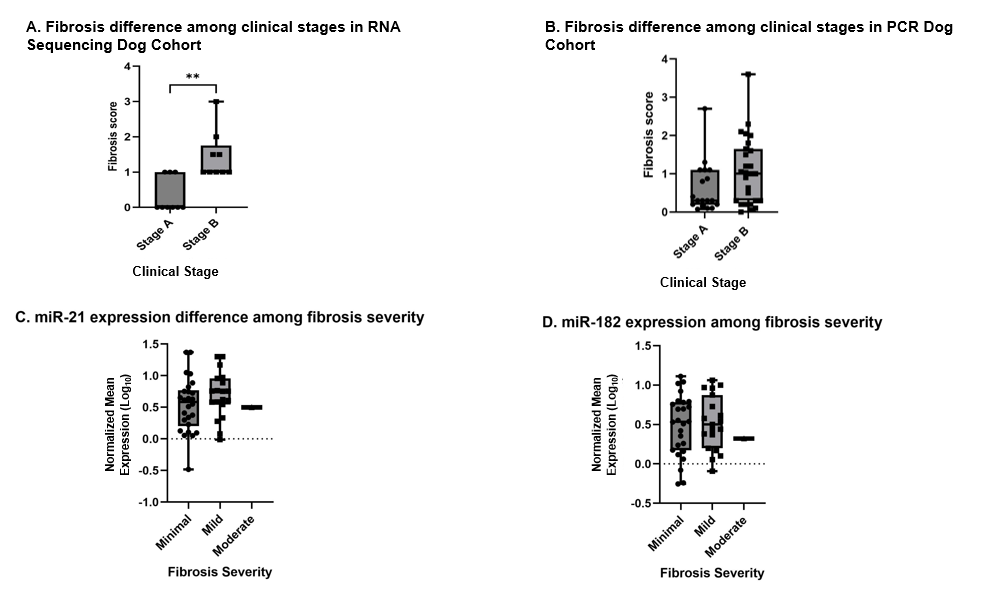


Supplementary Figure S4. Fibrosis difference among expression in dogs used in RNAseq and qRT-PCR analysis and qRT-PCR expression of miRs- 21 and 182 across fibrosis status. The median expression and individual values are displayed on the boxplot. Bars represent the upper, middle, and lower quartiles. Boxplots of fibrosis, distribution for dogs whose samples were used for the small RNA-seq analysis (A) versus the qRT-PCR dataset (B) among different azotemic statuses. Boxplots of miR-21 (C) and miR-486 (D) expression for dogs with minimal to mild fibrosis versus moderate to severe fibrosis. Stage A: sCr < 1.4 mg/dl or appropriately low for the breed and with biopsy findings limited to minimal to mild tubulointerstitial (TI) fibrosis. Stage B: 1.4 ≤ sCr ≤ 5 mg/dl) or sCr < 1.4 mg/dl but inappropriately high for the breed and biopsy results demonstrating significant TI fibrosis (moderate to severe). Mann Whitney U test was used in a pair-wise manner to compare the median of each category. *P < .05; **P < .01; ***P < .001.
